# The Physician’s Leisure Library: I. Bright’s Disease; II. The Modern Treatment of Diseases of the Kidneys

**Published:** 1889-03

**Authors:** 


**The Physician's Leisure Library. I. Bright's Disease.** By ALFRED L. LOOMIS, M. D., LL. D., Professor of Pathology and Practice of Medicine, New York University Medical College; Visiting Physician to Bellevue Hospital; Consulting Physician to St. Luke's, Mount Sinai Hospitals, etc., etc. George S. Davis, Detroit. 1888.

**II. The Modern Treatment of Diseases of the Kidney.** By PROFESSOR DUJARDIN-BEAUMETZ, Member of the Academy of Medicine and the Council of Hygiene and Salubrity of the Seine; Editor of the *Bulletin Général de Therapeutique*, Paris, France. Translated from the fifth French Edition by E. P. HURD, M. D., Newburyport, Mass. Detroit: George S. Davis. 1888.

These two books are a continuation of the most excellent series known as "The Physician's Leisure Library," for which the profession has to thank the enterprising publisher.

The first, by Dr. Loomis upon "Bright's Disease," is a scholarly exposition of the most modern thought upon a subject regarding which every practitioner must possess enlightened views. Our civilization is such as to engender maladies that were comparatively unknown to the ancients, and to even physicians of the last century. The function of the kidney becomes impaired by our present methods and habits of life, perhaps even more frequently than any other organ, and it is requisite that the earliest indication thereof shall be recognized by the average physician. Hence, such a book as this, written in his interest particularly, becomes of great value, and it is gratifying that authors and publishers are to be found both competent and willing to place such literature within the reach of the humblest purse. We could more strongly commend it had we space to say all that it deserves.

In the second, by Prof. Dujardin-Beaumetz, we have a most charming résumé of the treatment of the commoner forms of kidney disease, from the pen of an advanced thinker and a graphic writer. It is a most satisfactory little volume in every way, and these two compliment each other in a most fitting manner. They should be studied together to be appreciated; for then they may be contrasted, compared, and critically digested, with profit, and, we venture to say, without regret.

